# Species-specific climate Suitable Conditions Index and dengue transmission in Guangdong, China

**DOI:** 10.1186/s13071-022-05453-x

**Published:** 2022-09-27

**Authors:** Xinting Lu, Hilary Bambrick, Francesca D. Frentiu, Xiaodong Huang, Callan Davis, Zhongjie Li, Weizhong Yang, Gregor J. Devine, Wenbiao Hu

**Affiliations:** 1 School of Public Health and Social Work, Queensland University of Technology, Brisbane, Australia; 2 National Centre for Epidemiology and Population Health, The Australian National University, Canberra ACT, Australia; 3 Centre for Immunology and Infection Control, School of Biomedical Sciences, Queensland University of Technology, Brisbane, Australia; 4 Division of Infectious Disease, Key Laboratory of Surveillance and Early Warning of Infectious Disease, Chinese Centre for Disease Control and Prevention, Beijing, China; 5 School of Population Medicine & Public Health, Chinese Academy of Medical Science, Peking Union Medical College, Beijing, China; 6 Mosquito Control Laboratory, QIMR Berghofer Medical Research Institute, Brisbane, Australia

**Keywords:** Dengue transmission, Suitable conditional index, *Aedes aegypti*, *Aedes albopictus*, Interactive effect

## Abstract

**Background:**

Optimal climatic conditions for dengue vector mosquito species may play a significant role in dengue transmission. We previously developed a species-specific Suitable Conditions Index (SCI) for *Aedes aegypti* and *Aedes albopictus*, respectively. These SCIs rank geographic locations based on their climatic suitability for each of these two dengue vector species and theoretically define parameters for transmission probability. The aim of the study presented here was to use these SCIs together with socio-environmental factors to predict dengue outbreaks in the real world.

**Methods:**

A negative binomial regression model was used to assess the relationship between vector species-specific SCI and autochthonous dengue cases after accounting for potential confounders in Guangdong, China. The potential interactive effect between the SCI for *Ae. albopictus* and the SCI for *Ae. aegypti* on dengue transmission was assessed.

**Results:**

The SCI for *Ae. aegypti* was found to be positively associated with autochthonous dengue transmission (incidence rate ratio: 1.06, 95% confidence interval: 1.03, 1.09). A significant interaction effect between the SCI of *Ae. albopictus* and the SCI of *Ae. aegypti* was found, with the SCI of *Ae. albopictus* significantly reducing the effect of the SCI of *Ae. aegypti* on autochthonous dengue cases. The difference in SCIs had a positive effect on autochthonous dengue cases.

**Conclusions:**

Our results suggest that dengue fever is more transmittable in regions with warmer weather conditions (high SCI for *Ae. aegypti*). The SCI of *Ae. aegypti* would be a useful index to predict dengue transmission in Guangdong, China, even in dengue epidemic regions with *Ae. albopictus* present. The results also support the benefit of the SCI for evaluating dengue outbreak risk in terms of vector sympatry and interactions in the absence of entomology data in future research.

**Graphical Abstract:**

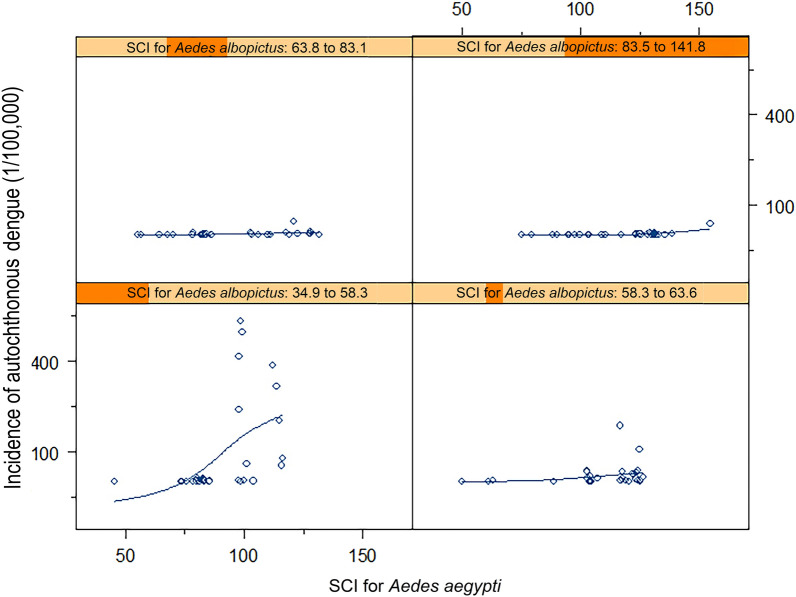

**Supplementary Information:**

The online version contains supplementary material available at 10.1186/s13071-022-05453-x.

## Background

The worldwide incidence of dengue has risen rapidly in recent decades. According to model estimates, 390 million dengue virus infections occur yearly [1] and 3.9 billion people are at risk of infection [2]. The number of dengue cases reported to the WHO have climbed from 505,430 in 2000 to over 2.4 million in 2010 and 5.2 million in 2019 [3]. To date, dengue is the most prevalent human arboviral infection worldwide. Dengue is an arthropod-borne virus (arbovirus) infection caused by four dengue virus (DENV) serotypes (DENV-1, -2, -3 and -4), with short-lived cross-protection between serotypes [4]. DENV is carried and spread by *Aedes* mosquito species, among which *Aedes aegypti* is the principal dengue vector that transmits the virus and causes epidemics. Other *Aedes* mosquito species known to be vectors of dengue include *Ae. albopictus*, *Ae. polynesiensis* and *Ae. scutellaris*; however, the latter two species are considered to have a limited capacity to serve as dengue vectors [5]. *Aedes aegypti* and *Ae. albopictus*, once zoophilic forest species from sub-Saharan Africa and Asia, respectively, are now widespread globally due to the rapid growth of the human population, intensified international travel in the last century and, more recently, climate change [6, 7].

*Aedes aegypti* is well-adapted to urban areas in tropical regions, while *Ae. albopictus* can thrive in peridomestic and rural habitats in tropical, subtropical and temperate climates [8]. In China, *Ae. aegypti* was once only present in the areas of the tropical zone below 22°N, such as Hainan Island, the Leizhou peninsula in Guangdong province and the coastal area along Beibu Gulf, Guangxi province. However, since the beginning of the present century, *Ae. aegypti* has also been found in some bordering counties in Yunnan province, located between 22°N and 25°N [9]. In contrast, *Ae. albopictus* has a wide distribution in both tropical and temperate regions of China, ranging from 41°N to the country’s southern reaches [10]. Based on historical climatic data from the last decade, most areas where *Ae. albopictus* has become established had an annual mean temperature > 11 °C and annual mean precipitation > 500 mm [11].

*Aedes aegypti* is considered to be the principal dengue vector and has contributed to a global resurgence of dengue epidemics in the past three decades, supported by evidence that all major epidemics of dengue have occurred only in areas where *Ae. aegypti* is found [12]. Conversely, *Ae. albopictus* is considered to play a relatively minor role in the transmission of DENV compared to *Ae. Aegypti* and is not considered an ‘efficient’ dengue epidemic vector [12, 13]. In China, dengue outbreaks in Hainan province in 1980 and 1985–1986 and in Xishuangbanna of Yunnan province in 2013 were typically attributed to *Ae. aegypti* [14, 15]. However, outbreaks, largely of classical dengue (i.e. few cases of severe disease), in Guangdong, Fujian and Zhejiang provinces between 2004 and 2010 were caused solely by *Ae. albopictus* in the absence of *Ae. aegypti* [16, 17]. In 2014, China’s largest dengue outbreak recorded to date (or at least since dengue became a notifiable disease in 1989) occurred in Guangdong, where *Ae. albopictus* appears to be the sole dengue vector [18].

A variety of factors affect the spatial and temporal dynamics of mosquito populations and therefore of dengue fever transmission patterns [19]. Temperature, rainfall and humidity affect the emergence and viability of eggs, and the size and longevity of adult mosquitoes and their dispersal [20]. Unplanned urbanisation, high population density and overcrowding favour the spread of mosquito breeding sites and infection [19]. The WHO recommends that many dengue-endemic nations use vector surveillance to provide a quantitative evaluation of fluctuations in the number and geographical distribution of dengue vector populations, with the purpose of forecasting outbreaks and assessing management options [21]. However, dengue transmission usually depends on a range of factors, including susceptible human host population(s), mosquito density and climate, rather than a fixed entomologic threshold [22]. Additionally, to date there has been minimal evidence of meaningful correlations between vector biomass indices and dengue transmission, which might be used to forecast epidemics consistently [23]. Furthermore, the geographical variability in the association between dengue vector abundance and transmission or outbreak occurrence is poorly understood, which suggests that vector population indices may have limited capacity in forecasting dengue transmission increases [24]. Therefore, other locality-specific data may have better potential as predictors.

Climate is a crucial environmental determinant of vector geographical distribution and vectorial capacity. Climate factors such as temperature and precipitation and their seasonal patterns can fundamentally affect mosquito population dynamics and individual features relevant to vector biology, such as development, reproduction and survival, and consequently DENV transmission patterns [19, 20, 25]. A sufficiently warm ambient temperature is also critical for virus replication and dissemination to the salivary glands in female mosquitoes. Conversely, cooler temperatures slow this viral amplification, and if the development time of the pathogen exceeds the lifespan of the infected mosquito, transmission cannot occur [8, 26]. Previous studies have estimated suitable temperatures that support dengue transmission [27–30]. Mordecai et al. synthesised existing empirical evidence on temperature suitability for vector and pathogen survival and used a mechanistic approach to ascertain suitable temperatures for vector capacity in dengue transmission. According to empirical data and probability models, suitable temperature ranges are between 17.05 °C and 34.61 °C for *Ae. aegypti* and between 15.84 °C and 31.51 °C for *Ae. albopictus* [31]. Based on these identified suitable temperature ranges for dengue vectorial capacity, we previously developed the Suitable Conditions Index (SCI) for *Ae. aegypti* and *Ae. albopictus*, respectively, to define their potential vector capacity. The SCI ranks geographical locations based on their climatic suitability for each vector species that transmits dengue and theoretically defines the spatial parameters for transmission [32]. By incorporating the SCI into an outbreak epidemiologic study, it is possible to investigate the potential value of SCI data for dengue transmission or outbreak prediction. In the study reported here, we used the autochthonous dengue case of the Guangdong dengue outbreak in 2014 to assess the potential of a climate-based SCI as a local dengue outbreak early warning index.

## Methods

### Study area

**Fig. 1:**
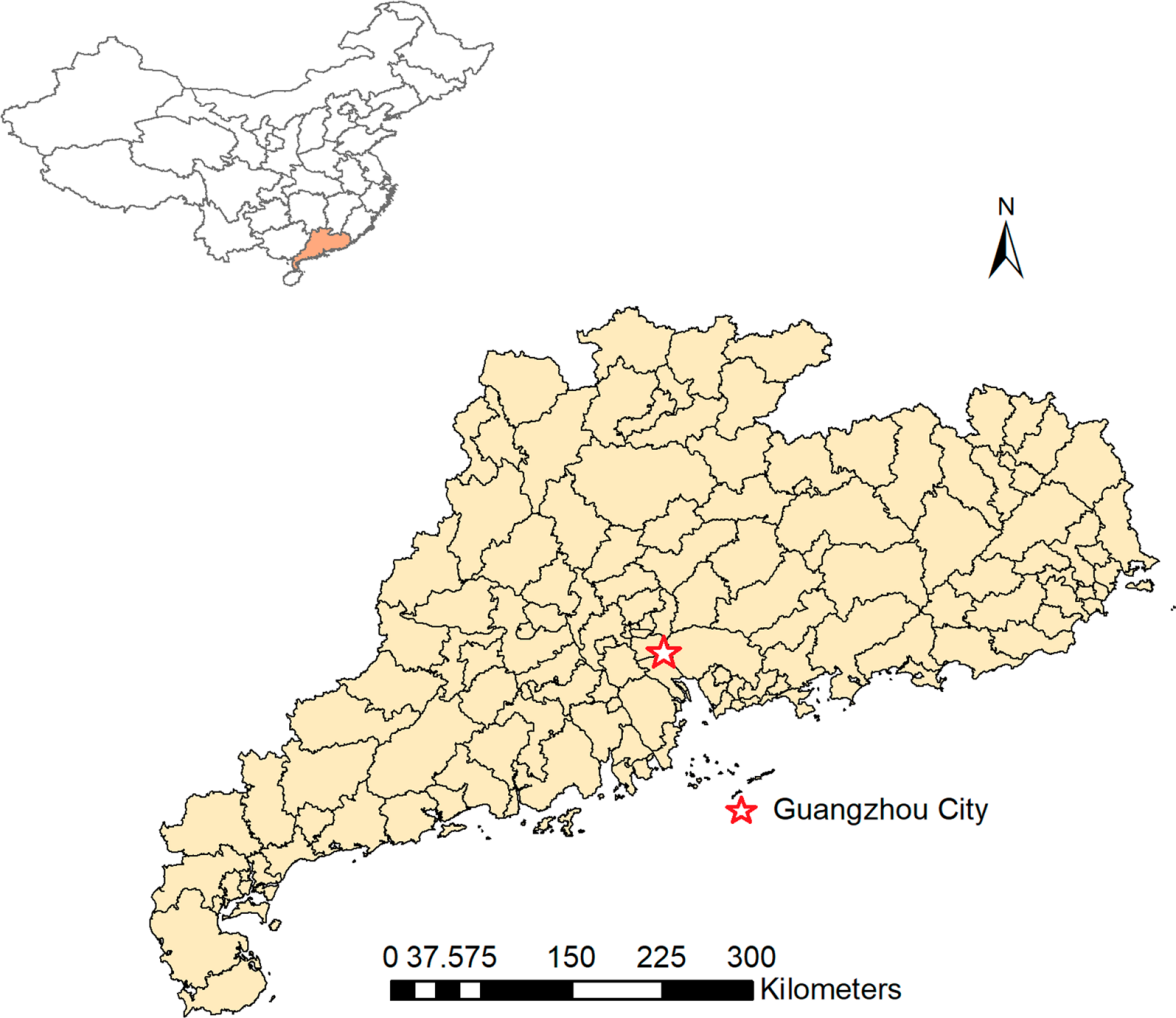
Study area and population density in Guangdong province, 2010

Guangdong (109°39′53″E to 117°18′51″E and 20°13′24″N to 25°31′10″N) Province, southern China, covers approximately 179,800 km^2^ in area. It is the most populous province in China, with 126.01 million inhabitants (7th National Population Census), accounting for 8.93% of Mainland China’s population [33] (Fig. 1). Guangdong has a humid subtropical climate, with short, mild and relatively dry winters and long, hot and very wet summers, influenced by the Asian monsoon season [34].

### Data collection

Data on autochthonous and imported dengue cases reported from Guangdong in 2014 were collected from the National Notifiable Infectious Diseases Reporting Information System. Dengue has been a statutory notifiable communicable disease in China since 1989 [35]. Dengue is diagnosed according to the national surveillance protocol with standardised case definitions, including clinically diagnosed and laboratory-confirmed dengue cases. An imported dengue case was defined when the patient had travelled abroad to a dengue-endemic country within 15 days of the onset of illness. In some cases, importation was defined based on laboratory results showing that the infecting DENV had genomic sequence similarity with viruses isolated from the putative source region where the patient had travelled [36]. In the absence of meeting the criteria for an imported case, a dengue case was considered to be autochthonous dengue case.

The SCI was developed in our previous study [32] to define climate suitability for *Ae. aegypti* and *Ae. albopictus*, respectively. Based on the optimal temperature ranges for vectorial capacity to transmit DENV identified by Mordecai et al. using robust mechanistic models [31], the SCI was defined as the product of the number of suitable months (where the monthly minimum and maximum temperatures fall within the determined temperature range) and the average monthly precipitation (as a weight). The input data for the SCI model were available on an annual interval and at the county scale. Therefore, we produced SCI values for each of *Ae. aegypti* and *Ae. albopictus* in 2014 for 124 counties/districts in Guangdong Province. In this study we used 2014 dengue outbreak data in Guangdong, which is the worst dengue outbreak in the last few decades in China. The dengue outbreak data across 124 districts/county levels provides a unique opportunity to identity the relationship between SCI and dengue cases at spatial distribution.

Normalised Difference Vegetation Index (NDVI) data were extracted from the Moderate Resolution Imaging Spectroradiometer (MODIS) website of the US National Aeronautics and Space Administration (NASA; https://www.nasa.gov/). NDVI data are used to characterise and monitor the global distribution of vegetation conditions and are often used to model global and regional climate [37]. We utilised MODIS Vegetation Indices (MOD13A3 product) version 6 data provided monthly at the 1-km spatial resolution, then aggregated annually and at the county scale [38]. In addition, human population data were obtained from the Sixth National Population Census data (2010), which is available from the National Bureau of Statistics of China (http://www.stats.gov.cn/enGliSH/).

### Negative binomial regression models

The negative binomial (NB) regression model was used to assess the relationship between autochthonous dengue cases and potential predictors, including SCI for each vector species, NDVI, human population density and the number of imported dengue cases. The NB regression model was also used to account for the over-dispersed count of dengue case (Table 1). A log-scale population was used as an offset to control for variation in human population size. Univariable and multivariable analyses were implemented successively. In the univariable analyses, we considered each potential predictor in an NB regression model to identify significant predictors. In a multivariable analysis, we examined the potential effect of significant predictors on dengue case occurrence (presented as incidence rate ratio [IRR]). Model goodness-of-fit was assessed by the Bayesian information criterion (BIC), with smaller values of BIC indicating a better fit.

We also examined the potential interaction between the SCI for *Ae. albopictus* and the SCI for *Ae. aegypti* (SCI-*Ae. aegypti* × SCI-*Ae. albopictus*). We assumed that the SCI was a proxy for vector density, with a high SCI value representing high vector density and vice versa. The interaction between the two vectors was examined by assessing how the effect of the SCI of a specific vector on autochthonous dengue case occurrence changed when the SCI of another vector changed. The difference in species-specific SCI (defined as ‘SCI-*Ae. Aegypti*—SCI-*Ae. albopictus’*) was also fitted to the NB regression model. As *Ae. albopictus* has a different niche to *Ae. aegypti* in terms of feeding preferences and, critically for the SCI, a different thermal range, it was reasonable to assume that a more considerable difference may indicate a reduced probability of ecological constraints between the two mosquito species. We therefore used the SCI difference to explore the correlation between the probability of ecological constraints and dengue transmission risk. The regression analysis was implemented in Stata/SE 16.0 (StataCorp, College Station, TX, USA) using “xtnbreg” (fixed-effects, random-effects and population-averaged negative binomial models, respectively) [39].

## Results

### Descriptive analyses

**Fig. 2:**
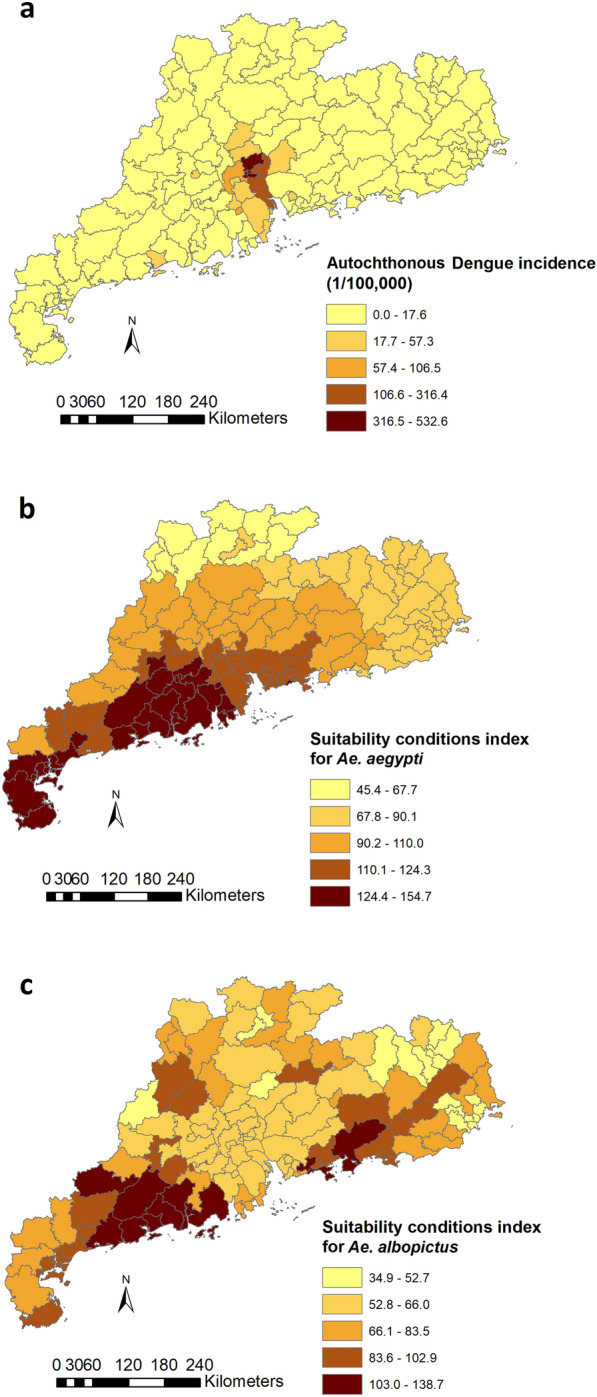
Geographical distribution of autochthonous dengue incidence (**a**), Suitable Conditions Index (SCI) for *Aedes aegypti* (**b**) and SCI for *Aedes albopictus* (**c**) at a county level in Guangdong Province, China, 2014

Summary statistics for autochthonous dengue cases, imported cases, NDVI, human population density, the SCI for *Ae. aegypti* and *Ae. albopictus*, respectively, are presented in Table 1. In 2014, there were 44,939 (44,862 autochthonous and 77 imported) dengue cases recorded from 107 of the 124 counties in Guangdong Province, with the number of all dengue cases per county/district ranging from 0 to 11,846 (mean number of cases per county/district: 362.41, standard deviation [SD]: 1388.57). The mean annual dengue incidence averaged 28.9 cases per 100,000 population, with the highest incidence (532.6/100,000) being reported from Baiyun District, followed by Liwan (497.3/100,000), Yuexiu (414.1/100,000) and Haizhu (385.2/100,000), all of which are in Guangzhou, the capital city of Guangdong (Fig. 2a).

**Table 1:** Summary statistics for autochthonous dengue cases, imported dengue cases, Normalised Difference Vegetation Index, human population density, Suitable Conditions Index for *Ae. aegypti* and *Ae. albopictus*, respectively, in 124 counties/districts of Guangdong Province, 2014

Variable	Mean	SD	Median	Minimum	Maximum
Autochthonous dengue cases	361.79	1387.31	10.50	0	11,837
Imported dengue cases	0.62	2.08	0	0	15
NDVI	185.40	28.42	193.22	94.78	255.00
Population density	2131.33	4008.42	502.35	76.91	21,686.58
SCI-*Ae.* *aegypti*	102.48	22.84	103.79	45.42	154.70
SCI-*Ae.* *albopictus*	71.79	20.69	63.60	34.92	138.68

*NDVI* Normalised Difference Vegetation Index, *SCI* Suitable Conditions Index, *SD* standard deviation

Guangdong was found to have the most suitable climatic conditions for dengue transmission for each vector (Fig. 2b, c). The SCI for *Ae. aegypti* was highest in the Leizhou peninsula below 22°N and in western coastal areas of the province between 22°N and 23°N, with a gradual reduction in suitability moving further north, and was lowest in the most northern part of the study area (Fig. 2b). The SCI for *Ae. albopictus* was less likely to be distributed evenly across low- to high-latitude areas (Fig. 2c). Regions with the highest SCI for both vectors overlapped in the western coastal areas below 23°N (Fig. 2b, c).

**Fig. 3:**
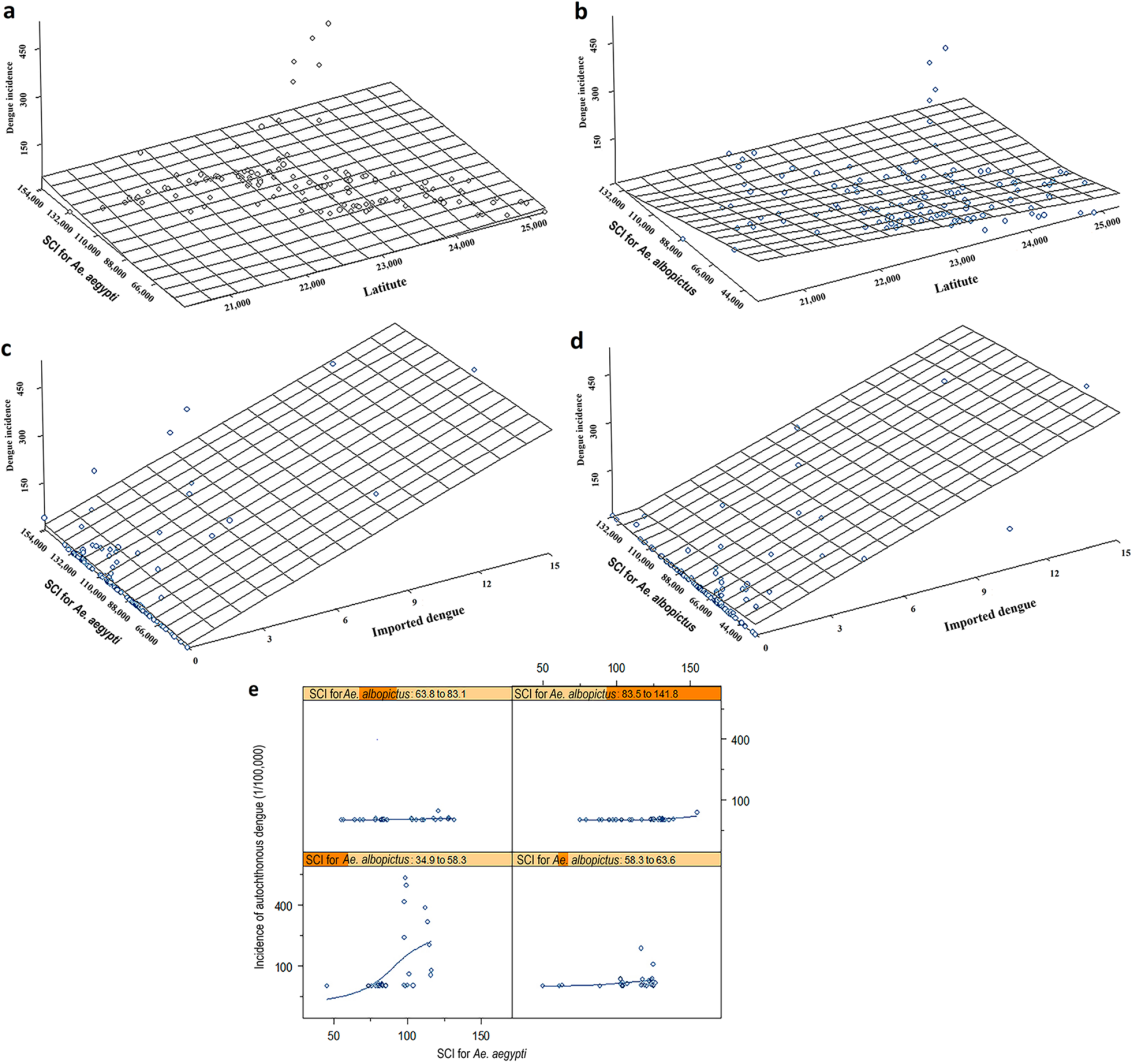
The relationship between autochthonous dengue incidence, latitude, imported dengue cases and Suitable Conditions Index (SCI) in Guangdong Province, China, 2014. Relationship between autochthonous dengue incidence, latitude and SCI for *Aedes aegypti* (**a**), relationship between autochthonous dengue incidence, latitude and SCI for *Aedes albopictus* (**b**), relationship between autochthonous dengue incidence and imported dengue cases and SCI for *Aedes aegypti* (**c**), relationship between autochthonous dengue incidence and imported dengue cases and SCI for *Aedes albopictus*** (d**), and relationship between autochthonous dengue incidence and SCI for *Aedes aegypti* over various levels of SCI for *Aedes albopictus* (**e**).

The relationship between autochthonous dengue incidence, latitude, imported cases and SCI for the two vector species is presented in Fig. 3a–d. The correlation between autochthonous dengue incidence and latitude was assessed against the SCI for *Ae. aegypti* and SCI for *Ae. albopictus*, respectively; a consistently higher value of autochthonous dengue incidence and SCI was found in areas at 23°N (Fig. 3a, b). The relationship between imported dengue cases, SCI for two mosquito vectors and autochthonous dengue cases indicated a strong relationship between autochthonous and imported dengue cases, which may remain independent over various levels of SCI for each vector species (Fig. 3c, d).

The relationship between autochthonous dengue incidence and SCI for *Ae. aegypti* was assessed when the SCI for *Ae. albopictus* was held constant at four levels ranging from low to high (34.9–58.1, 58.2–63.6, 63.8–84.5 and 85.5–141.8), and the results are shown in Fig. 3e. There was a moderate relationship between dengue incidence and SCI for *Ae. aegypti* when the SCI of *Ae. albopictus* was held constant at 34.9–58.1 (e.g. minimum to mean, with 1 SD) (Fig. 3e).

### Spearman’s correlation and bivariate Moran’s* I* statistic

Spearman’s correlation coefficients between any two variables considered in this study are presented in Table 2. There was a moderate relationship between autochthonous dengue incidence and the SCI for *Ae. aegypti* (*r*_*s*_ = 0.41, *P* = 0.000), while there was a weak correlation between incidence and the SCI for *Ae. albopictus* (*r*_s_ = -0.18, *P* = 0.000). There was a moderate correlation between autochthonous dengue incidence and difference in SCI (*r*_s_ = 0.53, *P* = 0.000). There was a moderate correlation between autochthonous dengue incidence and the other variables, including imported cases, NDVI and human population density. There was a moderate relationship between the two SCIs (*r*_*s*_ = 0.43, *P* = 0.000). We used bivariate Moran’s *I* [40] to further test the spatial relationship between the two SCIs. The global and local bivariate Moran’s *I* between the SCI for *Ae. albopictus* was significantly associated with the SCI for *Ae. aegypti* (Moran’s *I* = 0.426, *P* = 0.001) (Additional file 1: Figure S1).

**Table 2:** Correlation between autochthonous dengue annual incidence and exploratory variables

Variable	Autochthonous dengue incidence	SCI-*Ae*. *aegypti*	SCI-*Ae*. *albopictus*	Imported dengue	NDVI	Population density	Difference in SCI
Autochthonous dengue	1.00						
SCI-*Ae*. *aegypti*	0.41 (0.000)	1.00					
SCI-*Ae*. *albopictus*	− 0.18 (0.049)	0.43 (0.000)	1.00				
Imported dengue	0.50 (0.000)	0.18 (0.048)	− 0.20 (0.024)	1.00			
NDVI	− 0.56 (0.000)	−0.24 (0.007)	0.34 (0.000)	− 0.42 (0.000)	1.00		
Population density	0.64 (0.0000)	0.34 (0.000)	− 0.18 (0.044)	0.53 (0.000)	− 0.73 (0.000)	1.00	
Difference in SCI	0.53 (0.000)	0.53 (0.00)	− 0.46 (0.000)	0.32 (0.000)	− 0.51 (0.00)	0.46 (0.000)	1.00

Values in cells are Spearman’s rho (*r*_s_), with the* P*-value given in parentheses

### NB Regression models

The results of univariable analyses (Model I in Table 3) indicated that the SCI for *Ae. aegypti*, the difference between the SCI for *Ae. aegypti* and the SCI for *Ae. albopictus*, NDVI and imported cases were significant predictors of dengue transmission. The results of the multivariable models without the interaction terms (Models II and III) indicated that autochthonous dengue cases increased by 4% (IRR: 1.04, 95% confidence interval [CI] 1.02, 1.06) for a 1-unit increase in SCI for *Ae. aegypti*, but decreased by 3% (IRR: 0.97, 95% CI: 0.96, 0.99) for every 1-unit increase in SCI for *Ae. albopictus*. Autochthonous dengue cases increased by 28% (IRR: 1.28, 95% CI: 1.02, 1.60) and by 4% (IRR: 1.04, 95% CI: 1.02, 1.06) for every 1-unit increase in imported cases and difference in SCI, respectively. Dengue cases decreased by 3% (IRR: 0.97, 95% CI: 0.96, 0.99) for a 1-unit increase in NDVI. A significant interaction effect between the SCI of *Ae. albopictus* and the SCI of *Ae. aegypti* was found, with the SCI of *Ae. albopictus* significantly reducing the effect of the SCI of *Ae. aegypti* on autochthonous dengue cases (Table 3: Model IV). To avoid possible collinearity between NDVI and population density (*r*_s_ = −0.73, *P* = 0.000; Table 2; more importantly, the population density was not a significant predictor in multivariable models), the population density was excluded from the NB regression analyses.

**Table 3:** Incidence rate ratio and 95% confidence interval for potential predictors of autochthonous dengue case in Guangdong, 2014

Variable	Model I^a^	Model II^b^	Model III^c^	Model IV^d^
	IRR (95% CI)	IRR (95% CI)	IRR (95% CI)	IRR (95% CI)
SCI-*Ae. aegypti*	1.03 (1.02, 1.05)	1.04 (1.02, 1.06)	–	1.06 (1.03, 1.09)
SCI-*Ae. albopictus*	0.99 (0.97, 1.01)	0.97 (0.95, 0.99)	–	–
Difference in SCI-*Ae. aegypti* and SCI-*Ae. albopictus*	1.05 (1.04, 1.07)	–	1.04 (1.02, 1.06)	–
NDVI	0.95 (0.94, 0.97)	0.98 (0.96, 0.99)	0.98 (0.96, 0.99)	0.97 (0.96, 0.99)
Imported case	1.59 (1.31, 1.93)	1.28 (1.02, 1.61)	1.28 (1.02, 1.60)	1.28 (1.01, 1.61)
SCI-*Ae. aegypti* × SCI-*Ae. albopictus*^e^	–	–	–	0.99 (0.99, 0.99)^f^
BIC		1165.30	1162.67	1165.91

*BIC* Bayesian information criterion, *CI *confidence interval,* IRR* incidence rate ratio

^a^Univariable models

^b^Multivariable models with predictors being SCI-*Ae aegypti*, NDVI, Imported case

^c^Multivariable models with predictors being Difference in SCI-*Ae. aegypti* and SCI-*Ae. albopictus*, NDVI, Imported case

^d^Multivariable model III with interaction term SCI-*Ae. aegypti* × SCI-*Ae. albopictus*

^e^Interaction between the SCI for *Ae. albopictus* and the SCI for *Ae. aegypti*

^f^0.9998 (0.9996, 0.9999), *P* = 0.001

## Discussion

In the present study we investigated the association between vector species-specific SCI and autochthonous dengue cases in 2014, Guangdong province, China, and the value of SCI as a predictor of dengue outbreak risk in this geographical region. We found that the SCI was statistically associated with autochthonous dengue cases, implying that the SCI might be beneficial in forecasting the probability of local dengue transmission or outbreak occurrence. We also identified a statistically significant interaction between the SCI for *Ae. albopictus* and the SCI for *Ae. aegypti*, which invites the hypothesis that vector species interactions may also influence DENV transmission.

The SCI for *Ae. aegypti* was positively associated with autochthonous dengue transmission. However, dengue incidence was also negatively associated with the interactive effect between the SCI for *Ae. albopictus* and that for *Ae. aegypti*, which indicates that the effect of SCI for *Ae. aegypti* may change over the various levels of the SCI for *Ae. albopictus*. As shown in Fig. 3e, the region has a higher risk of dengue transmission if the region has a low SCI for *Ae. albopictus* but a high SCI for *Ae. aegypti*.

*Aedes aegypti* lives within or near human dwellings and tends to breed in urbanised ecological niches, whereas *Ae. albopictus* is more frequently found in rural areas and breeds outdoors. In addition, *Ae. aegypti* almost always feeds on humans, while *Ae. albopictus* usually bites humans and animals opportunistically [3]. These behavioural differences may indicate that *Ae. aegypti* has more opportunities to increase human-mosquito contact and thus increase the probability of DENV transmission. Although these two mosquito species are susceptible to infection by DENV, it the authors of a study conducted in Singapore reported that 6.9% of field-caught *Ae. aegypti* but only 2.9% of *Ae. albopictus* were DENV virus-positive [41]. *Aedes aegypti* is the primary dengue vector that can efficiently establish an outbreak. By contrast, *Ae. albopictus* plays a modest role in DENV transmission and is not an ‘efficient’ epidemic vector [12, 13]. The authors of another study conducted in Singapore reported that dengue-positive *Ae. aegypti* were detected 6 weeks before a dengue outbreak, but that infected *Ae. albopictus* did not appear until the number of cases was rising [42]. These differences in crucial characteristics, including behaviour and virus transmission capacity, may cause discrepancies in the roles of these two mosquito species in dengue outbreaks. There are still several significant gaps in our understanding of the correlations between climate suitability and virus transmission probability and intensity. Although temperature-dependent transmission models have been proven to be valuable for predicting vector spread and dengue transmission, most of these modelling studies found a very broad correlation of transmission occurring within a temperature range [25, 43]. Few models can sufficiently capture transmission dynamics to provide reliable predictions. Human mobility and behaviour, urban development and microclimates, aquatic habitat supply and vector control techniques may all impact dengue transmission [19, 44–49]. These characteristics may be as important as climate suitability in determining dengue transmission risk. Moreover, transmission dynamics and bionomic responses differ across vector species, mosquito populations and DENV serotypes [23, 50, 51]. While some potential confounders have been adjusted in our study, other elements may contribute to dengue transmission that we have not considered. Finally, whether or not primary mosquito vectors are present, optimal climatic conditions for dengue vector species may play a substantial role in an outbreak. This suggests the possibility that vector species are a prerequisite for initiating and establishing a dengue outbreak while suitable conditions may expand and maintain the outbreak through complicated effects on vector sympatry or interactions.

Although the SCI was not equivalent to mosquito species absence/presence or densities revealed only by entomological surveillance data, specific vector mosquitoes should only occur at a suitable habitat with favourable meteorological conditions. Our findings indicate the possibility that the SCI might be indirectly associated with autochthonous dengue cases through the variation in dengue vector population, which means that the SCI might be beneficial in the prediction of dengue transmission or outbreaks in the absence of entomological data. The SCI may therefore be meaningful in some dengue-endemic countries where entomological surveillance data are usually unavailable and, more importantly, where there has been no reliable indication of any consistent association between entomological indices and dengue cases [23].

Our results suggest a significant interaction between the SCI for *Ae. aegypti* (main effect) and the SCI for *Ae. albopictus*, which means the expected effect of the SCI for *Ae. aegypti* on autochthonic dengue cases may change over the various levels of the SCI for *Ae. albopictus*. Specifically, a 1-unit increase in the SCI for *Ae. aegypti* may indicate a higher risk of dengue in a region with a low SCI for *Ae. albopictus* than in a region with a high SCI for *Ae. albopictus*. Given that regions with a high SCI for each of these two *Aedes* vectors have some overlaps in Guangdong, the coexistence of these two vectors is theoretically possible. In areas sympatric for the two species, declining population and displacement of *Ae. aegypti* have been reported because of the superiority of *Ae. albopictus* in the competition for resources at the larval stage and asymmetric sterilisation at the adult stage following interspecific mating [52]. It has also been supposed that *Ae. aegypti* prefers to reproduce in areas devoid of *Ae. albopictus* [53]. As far as our knowledge, there is no sufficient evidence of vector competitiveness from field study in Guangdong. However, recent evidence from a laboratory experiment suggests competitive displacement is theoretically possible. In that laboratory experiment, larvae of the two species were mixed, allowed to emerge and then cycled through six generations, with the results showing that *Ae. aegypti* from the Leizhou peninsula were suppressed (a combination of generation time and abundance was used to judge competitiveness) by *Ae. albopictus* from Guangzhou but not by *Ae. albopictus* from other cities [54]. This finding warrants further exploration because the sympatric habit for the two species is expected to expand to additional areas in China [55].

We also observed that the difference in SCIs was positively associated with dengue transmission. The two *Aedes* species are usually distinct in terms of ecological environment, feeding preferences and, critically for the SCI, thermal range. Therefore, the high SCI for *Ae. aegypti* correlating with the low SCI for *Ae. albopictus* is meaningful, indicating that a larger difference in SCI may indicate less competition between the two mosquito species. In other words, areas that are optimally suitable for both species (i.e. low difference in SCI) may, somewhat counterintuitively, be associated with a decreased dengue transmission risk, potentially through competitive displacement of one species by the other.

There are several limitations to our study. The SCI is estimated on an annual basis, so it may not accurately capture seasonal variation and time-lag effects of climate factors in dengue case occurrence. A finer scale of SCI is thus needed in any subsequent spatiotemporal analysis in this geographical region. Furthermore, climate-based SCI may not accurately reflect dengue outbreak risk in tropical regions where temperature fluctuations have a weaker correlation with DENV transmission. In these tropical endemic countries, dengue outbreaks are most common in areas where multiple DENV serotypes are endemic and heterologous DENV infections are common [56, 57]. In tropical epidemic regions, the SCI should be validated against the locality-specific factor data to increase the predictive efficacy of the SCI. Although mosquito proliferation associated with high temperature and humidity, and the rainy season are major driving factors for dengue outbreaks, many socio-economic and environmental variables that are largely independent of climate, such as urbanisation and overcrowding, also facilitate the spread of dengue. These factors are not considered and accounted for in this study. However, as a first step, our SCI provides valuable insight into the possible relationship between optimal transmission environments and predictions of broad disease occurrence. To improve the prediction efficiency of the SCI, additional parameters, such as environmental variation (e.g. oviposition habitat availability, seasonal and daily temperature fluctuation) and socio-economic variables should be included. An enhanced SCI may be beneficial for extrapolating the possible geographical range of transmission beyond the existing environmental context (e.g. under climate change and for newly invading pathogens).

## Conclusions

The results of this study show that the climate-based SCI is beneficial for evaluating dengue outbreak risk and the potential effect of possible vector competition on dengue transmission. We suggest that the DENV may be more transmissible in a region with warmer weather conditions (high SCI for *Ae. aegypti*). The SCI for *Ae. aegypti* could be used to predict dengue transmission even in locations lacking *Ae. aegypti* but with *Ae. albopictus* present. Moreover, the SCI can be used to investigate the relative contributions of *Ae. albopictus* and *Ae. aegypti* to DENV outbreaks in the absence of entomology data in future research.

## Supplementary Information

**Additional file 1:** **Figure S1.** Bivariate Moran’s* I* scatter plot between the SCI for* Ae. aegypti* (original variable) and the SCI for* Ae. albopictus* (spatial lag as the second variable) in Guangdong, 2014 (**a**) and significant determinator (**b**).

## Data Availability

Data supporting the conclusions of this article are included within the article. In addition, the datasets used and analysed during the study are available from the corresponding author upon reasonable request.

## Abbreviations

BIC: Bayesian information criterion
DENV: Dengue virus
IRR: Incidence rate ratio
NB: Negative binomial
NDVI: Normalised Difference Vegetation Index
SCI: Suitable Conditions Index
