# Supplementary figures and images for: Species-specific climate Suitable Conditions Index and dengue transmission in Guangdong, China
Source: Parasit Vectors. 2022 Sep 27;15:342. doi: 10.1186/s13071-022-05453-x (PMC9516795; doi:10.1186/s13071-022-05453-x)

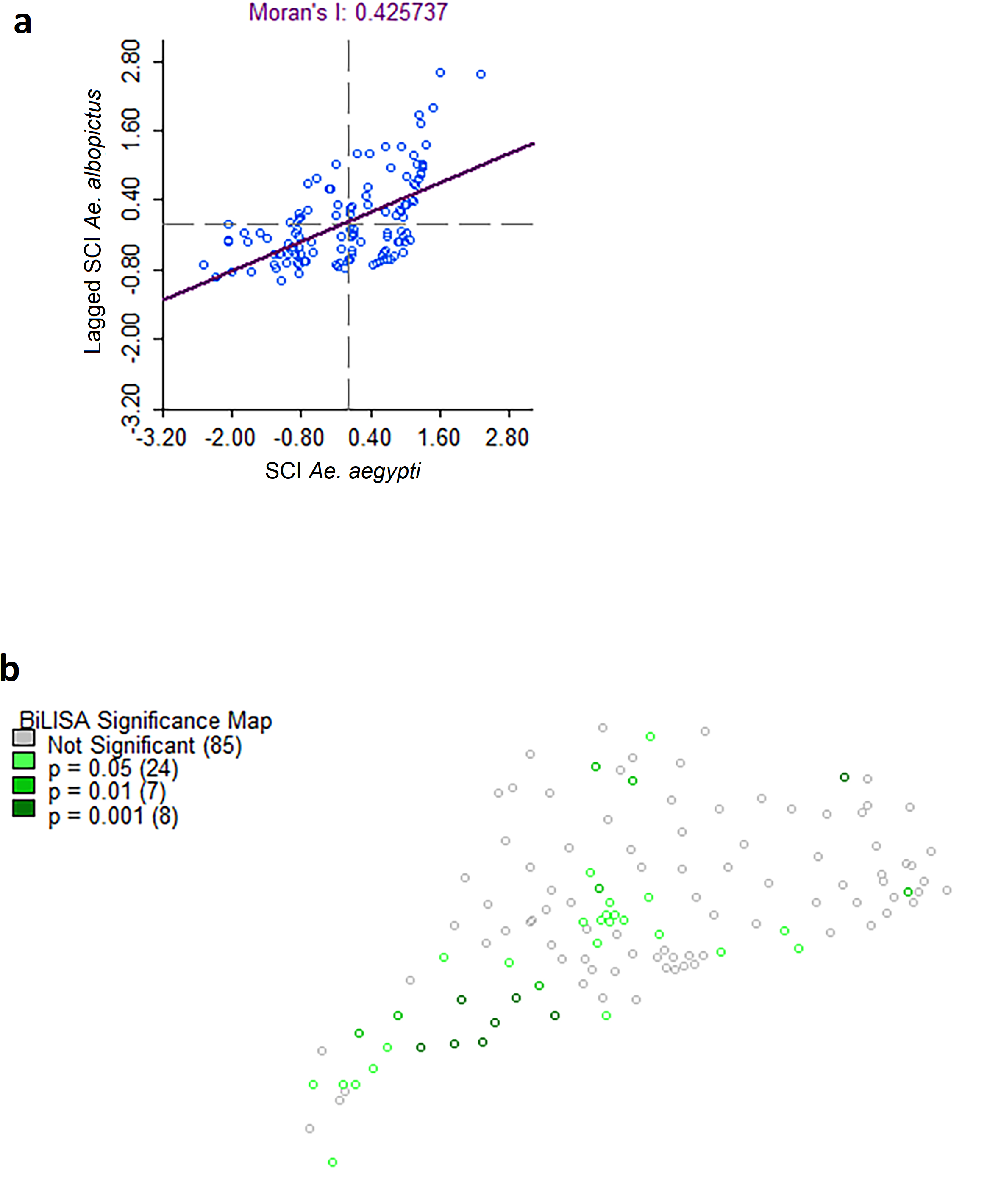

Supplement: Supplementary file 1 — Additional file 1: Figure S1. Bivariate Moran’s I scatter plot between the SCI for Ae. aegypti (original variable) and the SCI for Ae. albopictus (spatial lag as the second variable) in Guangdong, 2014 (a) and significant determinator (b). [file 13071_2022_5453_MOESM1_ESM.tif]
